# Human papillomavirus infection and risk of progression of epithelial abnormalities of the cervix.

**DOI:** 10.1038/bjc.1996.96

**Published:** 1996-02

**Authors:** C. B. Woodman, T. Rollason, J. Ellis, R. Tierney, S. Wilson, L. Young

**Affiliations:** Centre for Cancer Epidemiology, Christie Hospital NHS Trust, Manchester, UK.

## Abstract

The polymerase chain reaction has been used to determine the presence of human papillomavirus (HPV) 16 and HPV 18 DNA sequences in archival histological material removed from a cohort of untreated women with cervical epithelial abnormalities. The detection of HPV 16 or HPV 18 DNA sequences in the initial biopsy specimen was associated with a significantly increased risk of subsequent disease progression.


British Journal of Cancer (1996) 73, 553–556
© 1996 Stockton Press All rights reserved 0007–0920/96 $12.00

### Human papillomavirus infection and risk of progression of epithelial abnormalities of the cervix

CBJ Woodman[1], T Rollason[2], J Ellis[3], R Tierney[3], S Wilson[1] and L Young[3]

[1]*Centre for Cancer Epidemiology, Christie Hospital NHS Trust, Kinnaird Road, Withington, Manchester M20 4QL, UK;* [2]*Department of Pathology, Birmingham Maternity Hospital, Metchley Park Road, Edgbaston, Birmingham B15 2TG, UK;* [3]*CRC Department of Cancer Studies, University of Birmingham, Edgbaston, Birmingham B15 2TJ, UK.*

**Summary** The polymerase chain reaction has been used to determine the presence of human papillomavirus (HPV) 16 and HPV 18 DNA sequences in archival histological material removed from a cohort of untreated women with cervical epithelial abnormalities. The detection of HPV 16 or 18 DNA sequences in the initial biopsy specimen was associated with a significantly increased risk of subsequent disease progression.

**Keywords:** human papillomavirus 16; human papillomavirus 18; risk

Over the last 12 years a stream of imaginative and resourceful laboratory-based research has established an impressive set of oncogenic credentials for human papillomavirus (HPV). Two large population-based case–control studies have provided further support for its aetiological role in cervical neoplasia (Reeves *et al.*, 1989; Munoz *et al.*, 1992) and we now await the outcome of cohort studies in which longitudinal observations are made on subjects whose exposure status has been defined before the onset of disease. Such studies are under way but it will be some time before they are reported. An alternative strategy is to recruit a cohort of subjects who are likely to be at greater risk of developing cervical intraepithelial neoplasm (CIN) III and to determine the risk of progression in this cohort according to baseline HPV status. The aim of this study was to describe the association between the finding of HPV DNA sequences in the initial biopsies taken from a cohort of untreated patients with epithelial abnormalities of the cervix and the risk of subsequent progression of disease.

### Patients and methods

A randomised control trial undertaken some years ago provided a cohort of untreated patients with cervical epithelial abnormalities. The details of this trial have been outlined elsewhere (Woodman *et al.*, 1993). In brief, all women referred to the colposcopy clinics in the Birmingham and Midlands Hospital for Women between October 1983 and July 1985 for evaluation of cervical abnormality were included in the trial if: (a) the colposcopist making the first assessment considered them suitable for out-patient laser vaporisation and; (b) histological examination of a colposcopically directed punch biopsy revealed changes consistent with cervical human papillomavirus infection either alone or in association with CIN I or CIN II. Our criteria for selecting patients for laser vaporisation and the histological features used to diagnose HPV infection have previously been described (Byrne *et al.*, 1986).

Eligible patients were randomised into treatment and non-treatment groups. Untreated patients were monitored by regular cytological and colposcopic examination at intervals of 4 months and further histological sampling performed if the cytological or colposcopic findings suggested progression of disease. Progression of disease was defined as histological evidence of a change from HPV infection alone to CIN or an increase in the grade of CIN.

Correspondence: CBJ Woodman
Received 18 January 1995; revised 21 September 1995; accepted 28 September 1995

### *Laboratory methods*

When the randomised controlled trial was undertaken, routine determination of HPV DNA sequences in histological material was not feasible. It is now possible, however, using the polymerase chain reaction (PCR) to describe the presence of HPV DNA sequences in archival histological material (Imprain *et al.*, 1987; Shibata *et al.*, 1988; Resmick *et al.*, 1990). This allowed an assessment of the risk of progression in this cohort of untreated patients in relation to the presence of specific HPV DNA types in the baseline biopsy material. Unfortunately, the complete cohort of untreated patients could not be included in this analysis. Although all patients have been assessed in a dedicated research clinic by one observer, histological material removed during the course of the trial had been processed in one of two laboratories dependent upon the consultant to whom the patient had been initially referred. One laboratory routinely fixed specimens in Bouin's fluid and the other in formal saline. The use of the former fixative has been shown to inhibit the detection of HPV DNA sequences and these cases have been discarded. Baseline histological material from the remaining 93 cases was tested for the presence of HPV 16 or 18 DNA sequences using the PCR.

### *PCR methodology*

Paraffin-embedded tissue sections ($5 \times 10$ μM) were cut into a sterile Eppendorf tube and de-waxed using xylene. After centrifugation the xylene was removed, the tissue washed in 70% ethanol and the resultant pellet air dried. The tissue was then resuspended in 500 μl of 1 × PCR buffer (10 mM Tris-HCl, 1.5 mM magnesium–chloride, 50 mM potassium chloride, 0.1 mg $ml^{-1}$ gelatine, pH 8.3) containing 100 μg $ml^{-1}$ proteinase K and 0.5% Tween 80 at 55°C for 60 min, then incubated at 94°C for 10 min. A 10 μl aliquot of this preparation was then amplified in a 100 μl reaction as previously described (Tierney *et al.*, 1993) using L1 consensus oligonucleotide primers (Bauer *et al.*, 1991). The PCR consisted of primer extension for 2 min at 70°C, denaturation for 30 s at 94°C and reannealing for 90 s at 45°C. This was repeated for 40 cycles and the resulting amplified products were separated on a 3% agarose gel. The type specificity of the amplified products was assessed by Southern blotting followed by hybridisation with either an HPV 16-specific or an HPV 18-specific internal oligonucleotide probe. These probes were end labelled with 32 phosphorus using T4 polynucleotide kinase. The presence of amplifiable DNA in all extracted cases was confirmed using primers PC03 and PC04 specific for the human B-globin gene (Saiki *et al.*, 1985).

*Statistical methods*

Actuarial curves for the time to progression were drawn using the method of Kaplan and Meier and compared using the log-rank test (Kaplan and Meier, 1958). These curves were used when comparing the proportion of patients with progressive disease at 48 months in the HPV-positive and HPV-negative groups. Hazard ratios were also calculated to describe the magnitude of the association of baseline HPV status for each level of histological abnormality; 95% confidence intervals were constructed round each of these estimates (Machin and Gardner, 1988).

### Results

A total of 47 (51%) of the 93 subjects were found to have HPV 16 and/or 18 DNA sequences in their baseline biopsy specimens; 39 had HPV 16 alone; two HPV 18 alone and six both HPV 16 and 18. The remaining 46 (49%) subjects did not have HPV 16 or 18 DNA sequences in their baseline biopsy specimen and are hereafter referred to as HPV-negative. The mean age of HPV-positive subjects was 31.3 years (s.d. 7.7) and HPV-negative subjects 29.5 years (s.d. 7.5).

Of the 47 HPV-positive subjects, 21 (45%) had a histological diagnosis of HPV infection alone, 12 (26%) also had CIN I and 14 (30%) CIN II. Of the 46 HPV-negative subjects, 24 (53%) had a histological diagnosis of HPV infection alone, 15 (33%) also had CIN I and seven (15%) CIN II.

Median follow-up for all subjects was 36 months (range 4–84 months), 15 subjects (seven HPV-positive and 8 HPV-negative) were eventually treated, although there was no evidence of disease progression. These cases have been censored at the date of treatment. The probability of disease progression by baseline HPV status is shown in Figure 1. Five HPV-positive subjects progressed to CIN I, five to CIN II and 18 to CIN III. Two HPV-negative subjects progressed to CIN I, four to CIN II and seven to CIN III. Four years following randomisation, the proportion of HPV-positive subjects progressing to more severe disease was 56% compared with 25% of the HPV-negative subjects (95% confidence on difference in proportions 10–48%). The risk of progression in the HPV-positive group compared with the negative group, expressed as an odds ratio (OR) was 2.3 (95% CI 1.23, 4.27) for all cases, 1.87 (95% CI 0.75, 4.08) for those with histological evidence of HPV infection alone, 3.05 (95% CI 0.8, 11.65) for subjects with CIN I and 1.77 (95% CI 0.6, 5.7) for subjects with CIN II. When progression to CIN III was used as the sole study end point the risk of progression was found to be significantly greater in HPV-positive subjects ($P=0.015$) (Figure 2).

Number at risk

| | | | | | | |
|---|---|---|---|---|---|---|
| NPV -ve | 46 | 33 | 22 | 15 | 11 | 6 |
| NPV +ve | 47 | 32 | 20 | 9 | 7 | 2 |

**Figure 1** Comparison of rates of progression by baseline HPV 16 or 18 status.

Number at risk

| | | | | | | |
|---|---|---|---|---|---|---|
| HPV -ve | 46 | 33 | 22 | 15 | 11 | 6 |
| HPV +ve | 47 | 32 | 20 | 9 | 7 | 2 |

**Figure 2** Comparison of rates of progression to CIN III by baseline HPV 16 or 18 status

### Discussion

This study suggests that the presence of HPV 16 or 18 DNA sequences in baseline histological material taken from women with minor epithelial abnormalities of the cervix is associated with an increased risk of disease progression. The magnitude and direction of this association were similar for each level of histological abnormality. The findings are consistent with a number of previous reports. Campion *et al.* (1986) described a prospective study of 100 women with recurrent mildly dyskaryotic smears; 58% of those positive for HPV 16 progressed to CIN III within 2 years as compared with 20% of those with HPV 6. Koutsky *et al.* (1992) recruited a cohort of 241 women with negative cytology who were attending a sexually transmitted disease clinic; those who were HPV 16 or 18 positive were significantly more likely to progress to CIN II/III (OR = 11, 95% confidence intervals 4.6, 26). Murthy *et al.* (1990) using a nested case control design within a large cohort study again confirmed a significant risk of progression associated with the presence of baseline HPV 16 or 18 DNA sequences (OR = 5.9, 95% confidence intervals 2.5–14.1).

All studies of the natural history of early cervical neoplasia share a number of methodological problems. The first relates to the possible misclassification of baseline disease status. This is more likely when baseline status is defined on the results of cytological examination, which aims for detection rather than diagnostic accuracy. Histological examination of cervical tissue is more likely to provide an accurate diagnosis but this cannot always be guaranteed, because the most abnormal part of the lesion may not have been sampled. It has been suggested that the removal of tissue for diagnostic purposes may influence the natural history of the disease (Barron and Richart, 1968). The likelihood of this occurring is, of course, dependent upon the volume of tissue removed. Before the introduction of colposcopy and the use of local destructive techniques large wedge biopsies were removed from the cervix for diagnostic purposes. These may have been sufficient in some circumstances to abort the disease process. It is an entirely different proposition to suggest that the small volume of tissue removed by a colposcopically directed punch biopsy is sufficient to ensure disease regression in a substantial number of cases. The investigator is nevertheless obliged to trade off the need for an accurate definition of baseline status against the possibility that the means necessary to achieve this may influence the disease process.

The next problem relates to the detection of disease progression. CIN is an asymptomatic condition and therefore progression cannot be measured in continuous time. It can only be suspected by periodic observations made at discrete intervals using cytological and colposcopic examination, and only confirmed or refuted by histological examination. There is inevitably an element of subjectivity in deciding when the results of cytological and colposcopic examinations merit further histological sampling.

The same techniques must be used to establish follow-up and baseline disease status. It is not, for example, acceptable to define baseline disease status on the results of colposcopically directed punch biopsy and outcome on the results of a loop biopsy or cone biopsy. The use of the latter techniques allow for the removal of substantially greater volumes of tissue that provide for a more precise topographical description of the severity of the lesion.

There is a further difficulty in deciding what change in disease severity constitutes evidence of disease progression. Intuitively, the discovery of a CIN III lesion in a patient found to have CIN I at baseline provides more persuasive evidence of progression than when a CIN II lesion is discovered after the initial diagnosis of CIN I. Alternatively, both examples could be construed as evidence of progression from a 'low-grade' (HPV/CIN I) to a 'high-grade' lesion (CIN II/III). It might also be argued that changes in disease status over a short period of time are more likely to reflect misclassification following sampling error rather than true progression. Unfortunately, this requires us to make prior assumptions about the tempo of disease progression. These difficulties would be reduced, but not abolished, if CIN III alone was used as the study end point.

When this study was initiated ethical considerations dictated that women be treated at the time of histological confirmation of any disease progression. This was almost certainly unnecessary, as some cases might still have undergone spontaneous regression of epithelial abnormality. Nevertheless, sufficient women in this series have progressed to CIN III without any detectable intermediate stage to reveal a significant association between baseline HPV status and progression to CIN III.

All of the above caveats relating to the definition of baseline disease status also apply to the definition of baseline virological status, which will be influenced by the detection system used, the material provided for analysis and the accuracy of the sampling technique.

In this study we have only considered the prognostic importance of finding HPV 16 and 18 DNA sequences. We did not test for the presence of HPV 6/11 sequences because there was no a priori reason to believe these types were associated with an increased risk of disease progression. A high prevalence of other 'high-risk' HPV types (31, 33, 35, 39, 45, 51 or 52) have been reported in some North American series but surprisingly infrequently in this country (Cuzick *et al.*, 1992; Schiffman *et al.*, 1993). This may, of course, merely reflect the assiduousness with which they have been sought. If we had tested for the presence of other 'high-risk' HPV DNA types, this might have accentuated the difference between the progression curves.

Given these uncertainties, how robust are the conclusions that can be drawn from any analysis of risk factors for disease progression? It is clear that misclassification of virological status and disease status at baseline and during follow-up will have occurred. It is important to decide if this misclassification is likely to be random or systematic. Random misclassification will merely reduce differences between groups and attenuate measures of association. Systematic misclassification is more serious. If, for example, prior knowledge of virological status were to influence baseline or more importantly follow-up disease status, as a result of more intensive follow-up of HPV-positive patients, then spurious conclusions might be drawn. However, if virological status, baseline disease and follow-up status are independently defined, then these errors are likely to be random and as such will only underestimate the true risk of progression associated with HPV status. In this study the technological developments necessary to determine virological status only became available some years after the clinical trial had been completed, and both pathologist and clinician were therefore blind to the baseline HPV status of the cases.

The next major concern relates to the possibility of confounding. The association of HPV status with progression may be confounded if HPV infection is also associated with another factor that is itself a risk factor for progression. Risk of disease progression may be associated with baseline disease status but this study revealed a consistent association with HPV status for each level of baseline abnormality. Size of lesion has also been described as a risk factor for disease progression but there is no evidence linking HPV status and size. One other study has shown that the risk of disease progression is associated with the finding of other sexually transmitted agents including HPV but this analysis confirmed HPV status as an independent risk factor (Koutsky *et al.*, 1992).

There is another more serious reservation that applies to all natural history studies that use CIN III as an end point. Although the presence of HPV 16/18 infection may accelerate progression to CIN III, not all cases of CIN III will progress to invasive cancer (Kiviat *et al.*, 1992). As we cannot yet distinguish those cases which will progress it might be unwise to infer from these data that HPV infection results in the inexorable progression of all CIN lesions to invasive cancer.

**Acknowledgement**
We would like to acknowledge the continuing support and collaboration of clinicians in the Birmingham and Midlands Hospital for Women.

### References

BARRON BA AND RICHART RM. (1968). A statistical model of the natural history of cervical carcinoma based on a prospective study of 557 cases. *J. Natl Cancer Inst.*, **6**, 1343–1353.

BAUER HM, TING Y, GREER CE, CHAMBERS JC, TASHIRO CJ, CHIMERA J, REINGOLD A AND MANOS MM. (1991). Genital human papillomavirus infection in female university students as determined by a PCR-based method. *J. Am. Med. Assoc.*, **265**, 472–477.

BYRNE P, WOODMAN CBJ, MEANWELL CA, KELLY K AND JORDAN JA. (1986). Koilocytosis and cervical human papillomavirus infection. *Lancet*, **1**, 205–206.

CAMPION MJ, McCANCE DJ, CUZICK J AND SINGER A. (1986). Progressive potential of mild cervical atypia: prospective cytological, colposcopic, and virological study. *Lancet*, **2**, 237–240.

CUZICK J, TERRY G, HO L, HOLLINGWORTH T AND ANDERSON M. (1992). Human papillomavirus type 16 DNA in cervical smears as predictor of high-grade cervical cancer. *Lancet*, **339**, 959–960.

IMPRAIN CC, SAIKI RK, ERLICH HA AND TEPLITZ RL. (1987). Analysis of DNA extracted from formalin-fixed, paraffin-embedded tissues by enzymatic amplification and hybridisation with sequence-specific oligonucleotides. *Biochem. Biophys. Res. Commun.*, **142,** 710–716.

KAPLAN EL AND MEIER P. (1958). Non-parametric estimation from incomplete observations. *J. Am. Stat. Assoc.*, **53,** 457–481.

KIVIAT NB, CRITCHLOW CW AND KURMAN RJ. (1992). Reassessment of the morphological continuum of cervical intraepithelial lesions: does it reflect different stages in the progression to cervical carcinoma? In *The Epidemiology of Human Papillomavirus and Cervical Cancer* IARC Scientific Publication. Munoz N, Bosch FX, Shah K and Meheus. pp. 59–66. IARC: Lyon.

KOUTSKY LA, HOLMES KK, CRITCHLOW SW, STEVENS CE, PAAVONEN J, BECKMAN AM, DEROUEN TA, GALLOWAY DA, VERNON D AND KIVIAT NB. (1992). A cohort study of the risk of cervical intraepithelial neoplasia grade 2 or 3 in relation to papillomavirus infection. *New Engl. J. Med.*, **327,** 1272-1278.

MACHIN D AND GARDNER MJ. (1988). Calculating confidence intervals for survival time analysis. *Br. Med. J.*, **296,** 1369-1371.

MUNOZ N, BOSCH FX, DE SANJOSE S, TAFUR L, IZARZUGAZA I, GILI M, VILADIU P, NAVARRO C, MARTOS C, ASCUNCE N, GONZALEZ LC, KALDOR JM, GUERRERO E, LORINCZ A, SANTAHARIA M, DE RUIZ PA, ARISTIZABAL N AND SHAH K. (1992). The causal link between human papillomavirus and invasive cervical cancer: a population-based case-control study in Columbia and Spain. *Int. J. Cancer*, **52,** 743-749.

MURTHY NS, SEHGAL A, SATYANARAYANA L, DAS DK, SINGH V, DAS BC, GUPTA MM, MITRA AB AND LUTHRA UK. (1990). Risk factors related to biological behaviour of precancerous lesions of the uterine cervix. *Br. J. Cancer*, **61,** 732-736.

REEVES WC, BRINTON LA, GARCIA M, BRENES MM, HERRERO R, GAITAN E, TENORIO P, DE BRITTON RC AND RAWLS WE. (1989). Human papillomavirus infection and cervical cancer in Latin America. *New Engl. J. Med.*, **320,** 1437-1441.

RESNICK RM, CORNELISSEN MT, WRIGHT DK, EICHINGER GH, FOX HS, TER-SCHEGGET J AND MANOS MM. (1990). Detection and typing of human papillomavirus in archival cervical cancer specimens by DNA amplification with consensus primers. *J. Natl Cancer Inst.*, **82,** 1477-1484.

SAIKI RK, SCHARF S, FALOONA F, MULLIS KB, HORN GT, ERLICH HA AND ARNHEIM N. (1985). Enzymatic amplification of Beta-globin genomic sequences and restriction site analysis for diagnosis of sickle cell anemia. *Science*, **230,** 1350-1354.

SCHIFFMAN MH, BAUER HM, HOOVER RN, GLASS AG, CADELL DM, RUSH BB, SCOTT DR, SHERMAN ME, KURMAN RJ, WACHOLDER S, STANTON CK AND MANOS MM. (1993). Epidemiologic evidence showing that human papillomavirus infection causes most cervical intraepithelial neoplasia. *J. Natl Cancer Inst.*, **85,** 958-964.

SHIBATA DK, ARNHEIM N AND MARTIN WJ. (1988). Detection of HPV in paraffin-embedded tissue using polymerase chain reaction. *J. Exp. Med.*, **167,** 225-230.

TIERNEY R, ELLIS J, WINTER H, KAURESHI H, WILSON S, WOODMAN CBJ AND YOUNG LS. (1993). PCR for the detection of HPV16 infection: the need for standardisation. *Int. J. Cancer*, **54,** 1-2.

WOODMAN CBJ, BYRNE P, KELLY KA AND HILTON C. (1993). A randomised trial of laser vaporisation in the management of cervical intraepithelial neoplasia associated with HPV infection. *J. Pub. Health Med.*, **15,** 327-331.


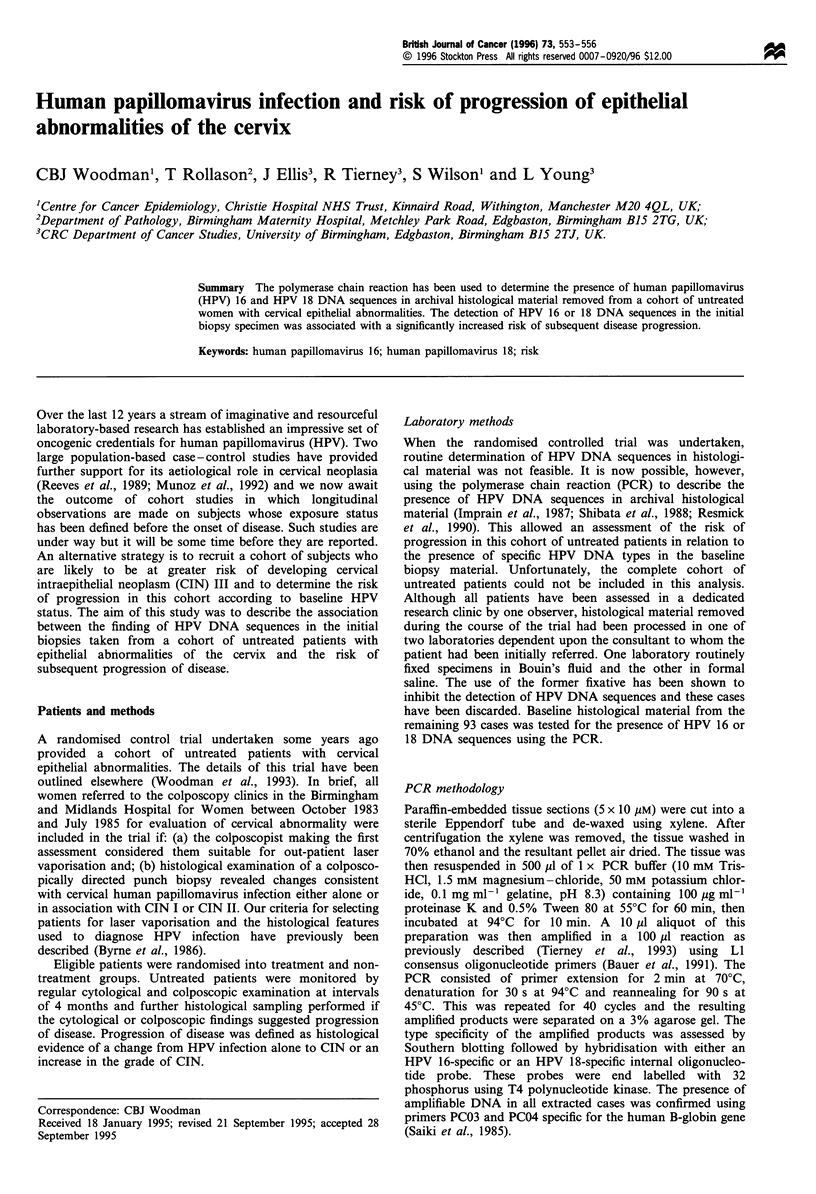

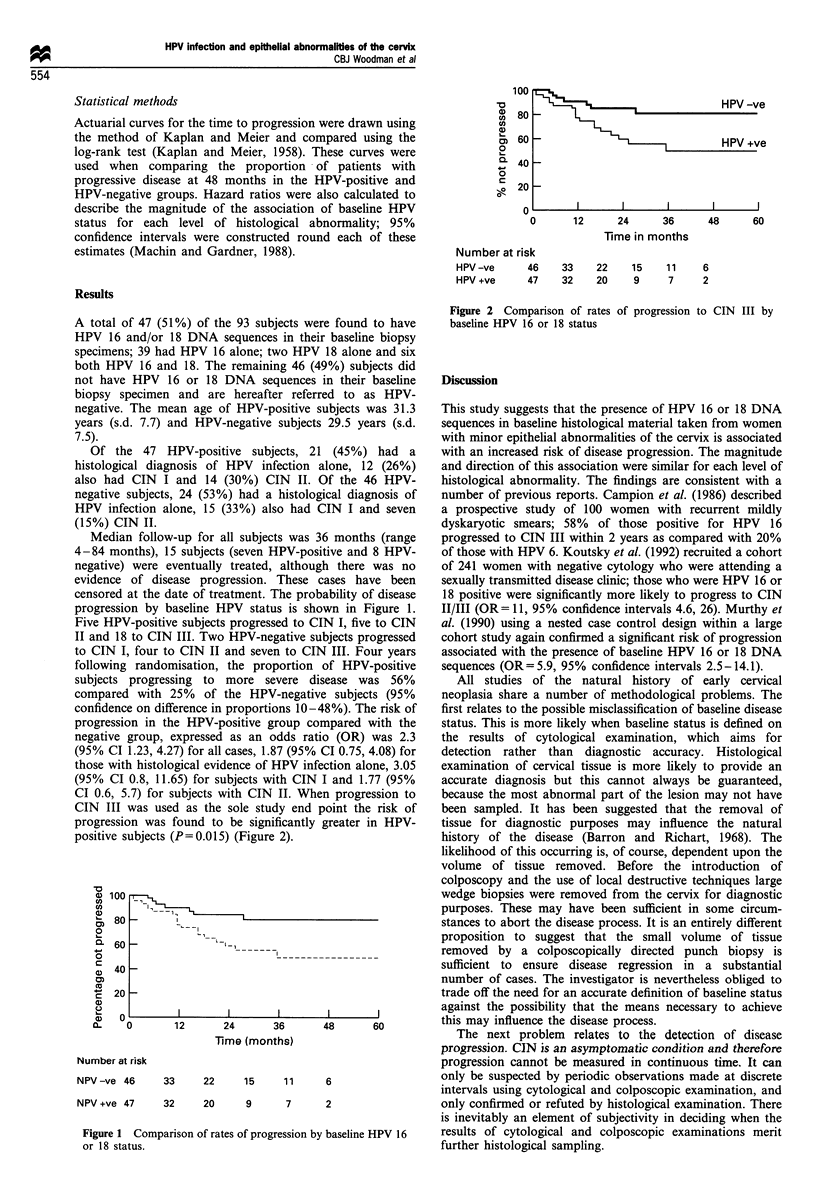

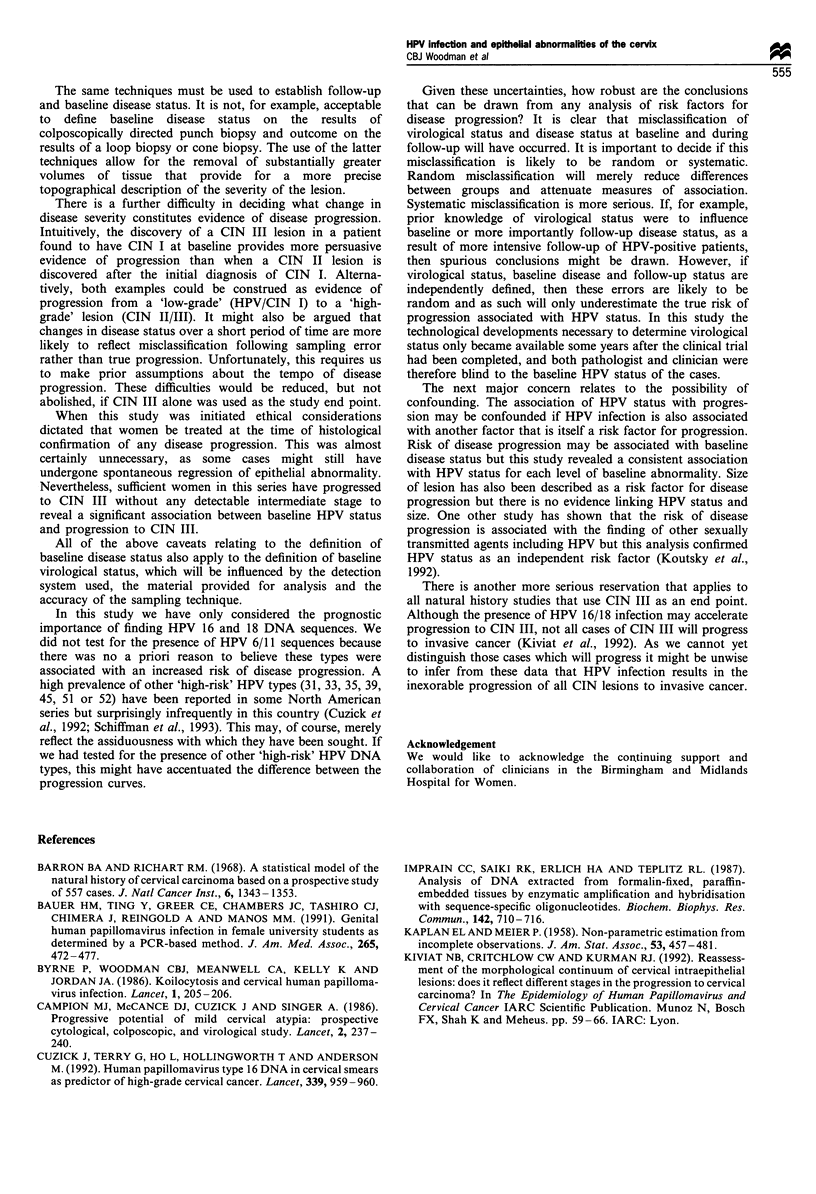

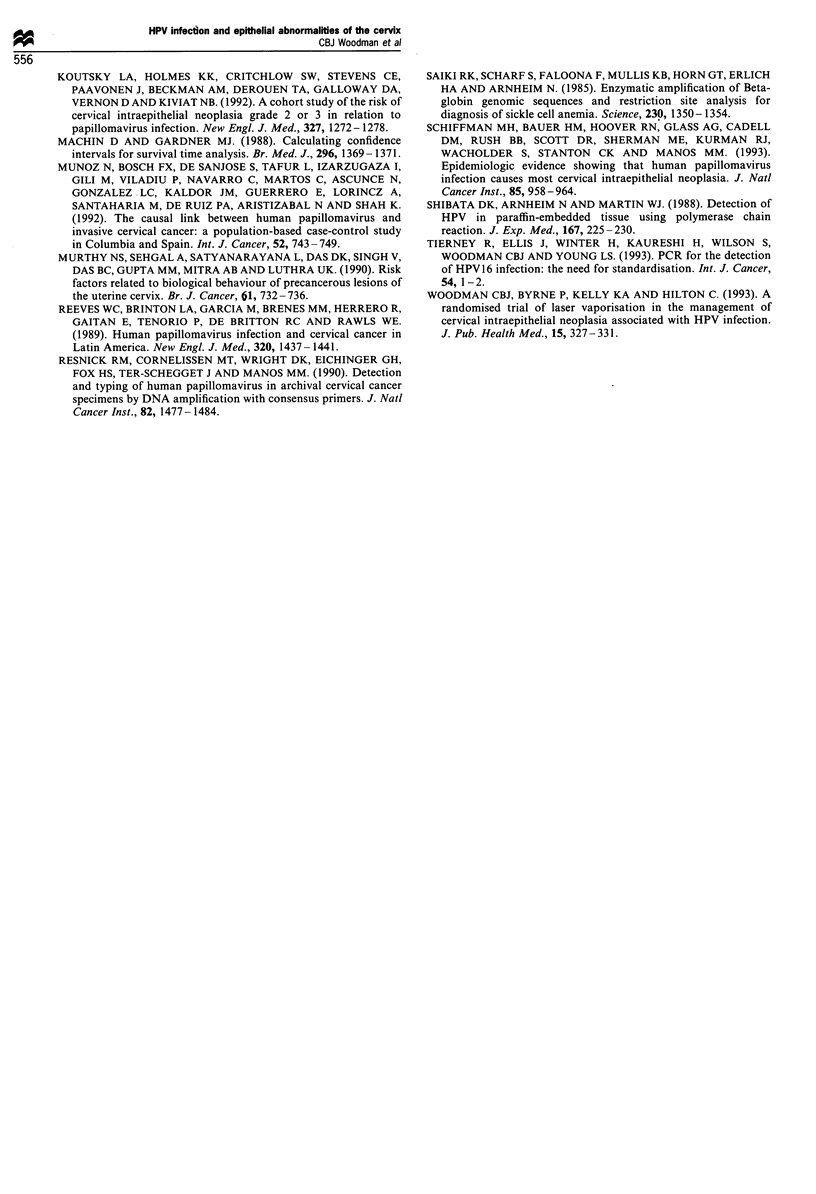


## References

[OCR_00500] Barron B. A., Richart R. M. (1968). A statistical model of the natural history of cervical carcinoma based on a prospective study of 557 cases.. J Natl Cancer Inst.

[OCR_00505] Bauer H. M., Ting Y., Greer C. E., Chambers J. C., Tashiro C. J., Chimera J., Reingold A., Manos M. M. (1991). Genital human papillomavirus infection in female university students as determined by a PCR-based method.. JAMA.

[OCR_00510] Byrne P., Woodman C., Meanwell C., Kelley K., Jordan J. (1986). Koilocytes and cervical human papillomavirus infection.. Lancet.

[OCR_00517] Campion M. J., McCance D. J., Cuzick J., Singer A. (1986). Progressive potential of mild cervical atypia: prospective cytological, colposcopic, and virological study.. Lancet.

[OCR_00521] Cuzick J., Terry G., Ho L., Hollingworth T., Anderson M. (1992). Human papillomavirus type 16 in cervical smears as predictor of high-grade cervical intraepithelial neoplasia [corrected].. Lancet.

[OCR_00528] Impraim C. C., Saiki R. K., Erlich H. A., Teplitz R. L. (1987). Analysis of DNA extracted from formalin-fixed, paraffin-embedded tissues by enzymatic amplification and hybridization with sequence-specific oligonucleotides.. Biochem Biophys Res Commun.

[OCR_00543] Kiviat N. B., Critchlow C. W., Kurman R. J. (1992). Reassessment of the morphological continuum of cervical intraepithelial lesions: does it reflect different stages in the progression to cervical carcinoma?. IARC Sci Publ.

[OCR_00552] Koutsky L. A., Holmes K. K., Critchlow C. W., Stevens C. E., Paavonen J., Beckmann A. M., DeRouen T. A., Galloway D. A., Vernon D., Kiviat N. B. (1992). A cohort study of the risk of cervical intraepithelial neoplasia grade 2 or 3 in relation to papillomavirus infection.. N Engl J Med.

[OCR_00558] Machin D., Gardner M. J. (1988). Calculating confidence intervals for survival time analyses.. Br Med J (Clin Res Ed).

[OCR_00106] Min J., Arganoza M. T., Ohrnberger J., Xu C., Akins R. A. (1995). Alternative methods of preparing whole-cell DNA from fungi for dot-blot, restriction analysis, and colony filter hybridization.. Anal Biochem.

[OCR_00571] Murthy N. S., Sehgal A., Satyanarayana L., Das D. K., Singh V., Das B. C., Gupta M. M., Mitra A. B., Luthra U. K. (1990). Risk factors related to biological behaviour of precancerous lesions of the uterine cervix.. Br J Cancer.

[OCR_00577] Reeves W. C., Brinton L. A., García M., Brenes M. M., Herrero R., Gaitán E., Tenorio F., de Britton R. C., Rawls W. E. (1989). Human papillomavirus infection and cervical cancer in Latin America.. N Engl J Med.

[OCR_00583] Resnick R. M., Cornelissen M. T., Wright D. K., Eichinger G. H., Fox H. S., ter Schegget J., Manos M. M. (1990). Detection and typing of human papillomavirus in archival cervical cancer specimens by DNA amplification with consensus primers.. J Natl Cancer Inst.

[OCR_00587] Saiki R. K., Scharf S., Faloona F., Mullis K. B., Horn G. T., Erlich H. A., Arnheim N. (1985). Enzymatic amplification of beta-globin genomic sequences and restriction site analysis for diagnosis of sickle cell anemia.. Science.

[OCR_00596] Schiffman M. H., Bauer H. M., Hoover R. N., Glass A. G., Cadell D. M., Rush B. B., Scott D. R., Sherman M. E., Kurman R. J., Wacholder S. (1993). Epidemiologic evidence showing that human papillomavirus infection causes most cervical intraepithelial neoplasia.. J Natl Cancer Inst.

[OCR_00603] Shibata D. K., Arnheim N., Martin W. J. (1988). Detection of human papilloma virus in paraffin-embedded tissue using the polymerase chain reaction.. J Exp Med.

[OCR_00612] Woodman C. B., Byrne P., Kelly K. A., Hilton C. (1993). A randomized trial of laser vaporization in the management of cervical intraepithelial neoplasia associated with human papilloma virus infection.. J Public Health Med.

